# Measurement-Device Independency Analysis of Continuous-Variable Quantum Digital Signature

**DOI:** 10.3390/e20040291

**Published:** 2018-04-17

**Authors:** Tao Shang, Ke Li, Jianwei Liu

**Affiliations:** 1 School of Cyber Science and Technology, Beihang University, Beijing 100083, China; 2 School of Electronic and Information Engineering, Beihang University, Beijing 100083, China

**Keywords:** measurement-device independency, quantum cryptographic protocols, quantum homomorphic signature, continuous variables

## Abstract

With the practical implementation of continuous-variable quantum cryptographic protocols, security problems resulting from measurement-device loopholes are being given increasing attention. At present, research on measurement-device independency analysis is limited in quantum key distribution protocols, while there exist different security problems for different protocols. Considering the importance of quantum digital signature in quantum cryptography, in this paper, we attempt to analyze the measurement-device independency of continuous-variable quantum digital signature, especially continuous-variable quantum homomorphic signature. Firstly, we calculate the upper bound of the error rate of a protocol. If it is negligible on condition that all measurement devices are untrusted, the protocol is deemed to be measurement-device-independent. Then, we simplify the calculation by using the characteristics of continuous variables and prove the measurement-device independency of the protocol according to the calculation result. In addition, the proposed analysis method can be extended to other quantum cryptographic protocols besides continuous-variable quantum homomorphic signature.

## 1. Introduction

Quantum cryptography is believed to be unconditionally secure because its security is ensured by physical laws rather than computational complexity. In virtue of no-cloning theorem and uncertainty principle, an attacker can neither distinguish between two non-orthogonal quantum states nor copy an unknown quantum state. Many quantum cryptographic protocols have been proposed based on this feature of quantum states and have been proved secure in both theoretical and experimental ways.

According to the fact that a quantum system has either a discrete spectrum or a continuous spectrum, quantum information can be classified into two categories, namely discrete variables and continuous variables. Discrete-variable quantum cryptographic protocols are more widely studied but are more expensive than continuous-variable ones. Continuous-variable quantum cryptography has gained much attention for practical advantages of low cost, high efficiency and compatibility with current optical fiber communication systems. Since continuous-variable quantum cryptographic protocols are very probable to be implemented in practice, such analysis which assumes all devices are perfect is insufficient to judge whether a protocol is truly secure or not. An attacker could exploit the loopholes of a device to successfully attack a protocol even though it is proved theoretically secure. To analyze the practical security of a quantum cryptographic protocol, the definition of device independency was proposed. If a protocol can complete its task securely, even if all devices are untrusted, which means some devices might be controlled by an attacker, it is called a device-independent (DI) protocol.

To date, research on device independency analysis only focuses on quantum key distribution (QKD) protocols. In 2006, Acin et al. [1] proposed the first device-independent quantum key distribution (DI-QKD) protocol and proved its security against individual attacks. Before long security analyses against collective attacks of DI-QKD protocols were proposed [2,3]. Since 2011, general formalisms for proving the security of DI-QKD protocols have been proposed [4,5], which can defend against the most general attacks. However, these analyses were proposed for discrete-variable QKD protocols, which means they cannot be directly applied to continuous-variable quantum cryptographic protocols.

As we know, in the continuous-variable setting, research focuses more on the measurement-device independency of a protocol rather than device independency, which only considers the independency of measurement devices. Measurement devices are the devices used for measuring quantum observables, such as beam splitter (BS) and homodyne detector. The concept of measurement-device independency was put forward by Lo et al. [6] in 2012. It can be regarded as a weakened version of device independency because it only considers the security loopholes of measurement devices. Compared to DI quantum cryptographic protocols, measurement-device-independent (MDI) quantum cryptographic protocols can achieve higher efficiency with practical implementation while not losing much security, so it is widely studied. To improve practicability and efficiency of QKD, several continuous-variable measurement-device-independent quantum key distribution (CV-MDI-QKD) protocols were proposed [7,8,9]. In recent years, study on measurement-device independency extends to other types of quantum cryptographic protocols other than QKD. In 2016, Wu et al. [10] proposed a CV MDI multipartite quantum communication protocol, which can implement both quantum cryptographic conference and quantum secret sharing. In 2018, Li et al. [11] tried to solve the practical problem of implementing scalable quantum networks and proposed a CV MDI quantum relay network with phase-sensitive amplifiers. Recently, towards estimating entanglement in a quantum network, MDI entanglement estimation schemes were proposed [12,13].

Since there exist different security problems for different protocols, device independency analysis of other continuous-variable quantum cryptographic protocols except QKD protocols should be explored. Continuous-variable quantum digital signature (CVQDS) [14,15,16] is a sufficiently studied technology in the field of continuous-variable quantum cryptography. It is an essential part of a secure continuous-variable quantum network so its device independency can affect the practical security of a network. However, there is no research on the measurement-device independency of CVQDS. Generally, CVQDS protocols are not device-independent because secret keys are directly passed to the device that generates signatures as a parameter, in which case an attacker can easily obtain the secret keys. Therefore, we assume the devices for quantum state preparation are trusted and perfect, and focus on analyzing the measurement-device independency of CVQDS.

The main contributions of our paper are:

(1) We analyze the measurement-device independency of a continuous-variable quantum digital signature scheme. We point out that a CVQDS protocol is measurement-device-independent if its error rate is negligible on condition that all measurement devices are untrusted. The error rate is composed of two aspects, i.e., probability of a forged signature passing verification and probability of a legal signature being denied. Calculation results show the continuous-variable quantum homomorphic signature scheme is measurement-device-independent.

(2) We simplify the calculation of error rate by means of utilizing the characteristics of continuous variables. The probability of a forged signature passing verification, which is related to the von Neumann entropy of the quantum system in the protocol, is calculated from the covariance matrices of Gaussian states instead of the trace of a infinite-dimensional density matrix. The probability of a legal signature being denied is equivalent to the probability that the absolute value of a random variable is greater than its standard deviation. Since the random variable follows the Gaussian distribution, the probability can be immediately obtained without calculation.

## 2. Related Works

### 2.1. Measurement-Device Independency

If a quantum cryptographic protocol can complete its task securely with untrusted measurement devices, it is called a measurement-device-independent protocol. To analyze the security of a quantum cryptographic protocol under the worst case, we assume measurement devices are prepared and controlled by an attacker and can work in the way that is most favorable to the attacker. Concretely, the assumptions are:

(1) An attacker can tamper and forge the output of measurement devices.

(2) An attacker can eavesdrop quantum channels by any means.

For simplicity, we call the above assumptions the MDI assumptions. In other words, if the task of a quantum cryptographic protocol is completed under the MDI assumptions, the protocol is measurement-device-independent.

To date, there are only achievements of MDI analysis for QKD protocols. The first MDI-QKD protocol was proposed by Lo et al. [6], which is a discrete-variable quantum cryptographic protocol. The security proof utilizes the monogamous nature of quantum entanglement and removes detector side-channel attacks while it is not a mathematical proof. In the same year, Ma and Razavi [17] proposed the alternative schemes for MDI-QKD using phase and path or time encoding. In the security analysis, the lower bound of secret key rate was calculated. A protocol is secure if its secret key rate is higher than the lower bound. In 2014, several CV-MDI-QKD protocols were proposed [7]. In the security analysis, the secret key rate of an equivalent one-way CVQKD model was calculated, which is the lower bound for the proposed protocol. Calculation was simplified by applying the theorem of the optimality of Gaussian collective attacks [18]. The analysis of other CV-MDI-QKD protocols [8,9] are similar in calculating the lower bound of secret key rate.

Obviously, we cannot directly calculate the secret key rate of a non-CVQKD protocol, so we should put forward a new method of analyzing its measurement-device independency.

### 2.2. Continuous-Variable Quantum Homomorphic Signature

In CVQDS protocols, there are usually at most three participants, i.e., a signer, a verifier and an arbitrator. Since the verifier and the arbitrator are assumed to be honest, the only untrusted party is the signer, so it seems easy to analyze measurement-device independency. Nevertheless, in 2017, Li et al. [19] proposed a continuous-variable quantum homomorphic signature (CVQHS) scheme, where an aggregator generates a homomorphic quantum signature for verifying the identities of multiple data sources. The aggregator has access to all quantum and classical data in the network, so the scheme probably will not be secure if an attacker takes control of the devices of the aggregator. The existence of an untrusted aggregator has posed a new challenge in analyzing the measurement-device independency of CVQDS.

**Figure 1:**
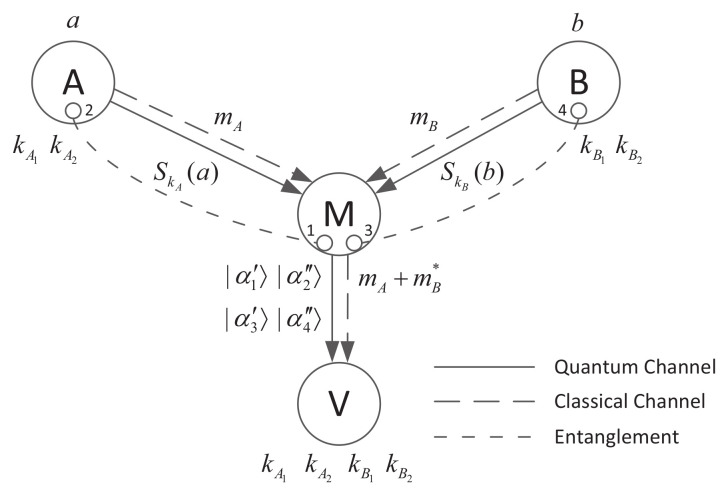
Basic model of CVQHS.

Li’s CVQHS scheme is based on continuous-variable entanglement swapping and provides additive and subtractive homomorphism. The basic model of the CVQHS scheme is shown in Figure 1. *A* and *B* are signers, *M* is an aggregator who aggregates the received signatures to generate two new signatures, and *V* is a verifier.

The CVQHS scheme is defined by a tuple of algorithms (Setup, Sign, Combine, Verify) and is briefly described as follows.

(1) Setup

Step 1. *A* shares two secret keys $k_{A_{1}}$ and $k_{A_{2}}$ with *V* by continuous-variable quantum key distribution. Meanwhile, *B* shares two secret keys $k_{B_{1}}$ and $k_{B_{2}}$ with *V*. The secret keys are real numbers.

Step 2. *M* prepares two pairs of entangled states, namely $(|\alpha_{1}\rangle ,|\alpha_{2}\rangle )$ and $(|\alpha_{3}\rangle ,|\alpha_{4}\rangle )$. Then, *M* sends $|\alpha_{2}\rangle$ to *A* and $|\alpha_{4}\rangle$ to *B*.

(2) Sign

Step 1. *A* signs its classical message *a* by displacing the quadratures of $|\alpha_{2}\rangle$. The signature of *A* is $S_{k_{A}}\left(a\right)=|\alpha_{2}+m_{A}+m_{k_{A_{1}}}+m_{k_{A_{2}}}\rangle =|{\alpha^{\prime }}_{2}\rangle$, where $m_{A}=a+ia$, $m_{k_{A_{1}}}=k_{A_{1}}+ik_{A_{1}}$, and $m_{k_{A_{2}}}=x_{k_{A_{2}}}+ip_{k_{A_{2}}}$. $x_{k_{A_{2}}}$ and $p_{k_{A_{2}}}$ are determined by the classical message and $k_{A_{2}}$:

(1) $$\left\{\begin{array}{cc} x_{k_{A_{2}}}=k_{A_{2}},p_{k_{A_{2}}}=0\; & if\;a+k_{A_{2}}\;is\;odd\\ x_{k_{A_{2}}}=0,p_{k_{A_{2}}}=k_{A_{2}}\; & if\;a+k_{A_{2}}\;is\;even \end{array}\right..$$

Similarly, *B* signs its classical message *b* by displacing the quadratures of $|\alpha_{4}\rangle$.

Step 2. *A* sends the signature $|\alpha_{2}^{\prime }\rangle$ and the classical message $m_{A}$ to *M*, while *B* sends the signature $|\alpha_{4}^{\prime }\rangle$ and the classical message $m_{B}$ to *M*.

(3) Combine

Step 1. *M* applies Bell detection on $|\alpha_{1}\rangle$ and $|\alpha_{3}\rangle$ and obtains the classical measurement results $x_{1}^{\prime }=\frac{1}{\sqrt{2}}\left(x_{1}+x_{3}\right)$ and $p_{3}^{\prime }=\frac{1}{\sqrt{2}}\left(p_{1}‒p_{3}\right)$.

Step 2. *M* mixes $|\alpha_{2}^{\prime }\rangle$ and $|\alpha_{4}^{\prime }\rangle$ at a 50:50 BS and obtains two new signatures

(2) $$\left\{\begin{array}{c} |{\alpha^{\prime \prime }}_{2}\rangle =|\frac{1}{\sqrt{2}}\left({\alpha^{\prime }}_{2}+{\alpha^{\prime }}_{4}\right)\rangle \\ |{\alpha^{\prime \prime }}_{4}\rangle =|\frac{1}{\sqrt{2}}\left({\alpha^{\prime }}_{2}‒{\alpha^{\prime }}_{4}\right)\rangle \end{array}\right..$$

Step 3. *M* sends the quantum states $|\alpha_{1}^{\prime }\rangle$, $|\alpha_{2}^{\prime \prime }\rangle$, $|\alpha_{3}^{\prime }\rangle$, $|\alpha_{4}^{\prime \prime }\rangle$ and the classical message $m_{A+B}=m_{A}+m_{B}^{*}$ to *V*.

(4) Verify

Step 1. *V* measures the *x* quadrature of $|\alpha_{2}^{\prime \prime }\rangle$ and the *p* quadrature of $|\alpha_{4}^{\prime \prime }\rangle$ by homodyne detection and obtains the measurement results $x^{\prime \prime }$ and $p^{\prime \prime }$.

Step 2. *V* measures the *x* quadrature of $|\alpha_{1}^{\prime }\rangle$ and the *p* quadrature of $|\alpha_{3}^{\prime }\rangle$ by homodyne detection and obtains $x_{1}^{\prime }$ and $p_{3}^{\prime }$. Then, *V* calculates $x_{V}=\sqrt{2}\left(x_{2}^{\prime \prime }‒\tau x_{1}^{\prime }\right)$ and $p_{V}=\sqrt{2}\left(p_{4}^{\prime \prime }‒\tau p_{3}^{\prime }\right)$, where τ is the transmissivity of quantum channels.

Step 3. *V* calculates *a* and *b* from the received classical message $m_{A+B}=m_{A}+m_{B}^{*}$. Then, *V* calculates $m_{k_{A_{2}\left(B_{2}\right)}}$, $x_{V}^{\prime }=a+k_{A_{1}}+x_{k_{A_{2}}}+b+k_{B_{1}}+x_{k_{B_{2}}}$, and $p_{V}^{\prime }=a+k_{A_{1}}+p_{k_{A_{2}}}‒b‒k_{B_{1}}‒p_{k_{B_{2}}}$ according to pre-shared secret keys. To verify the authenticity and integrity of the signatures, *V* calculates $H_{x}={\left(x_{V}‒\tau x_{V}^{\prime }\right)}^{2}$ and $H_{p}={\left(p_{V}‒\tau p_{V}^{\prime }\right)}^{2}$. If $H_{x}\leq H_{th}$ and $H_{p}\leq H_{th}$, *V* will confirm that $|\alpha_{2}^{\prime \prime }\rangle$ and $|\alpha_{4}^{\prime \prime }\rangle$ are the signatures of *M* and accept the classical messages *a* and *b*. Otherwise, *V* will deny the signatures. $H_{th}$ is the verification threshold.

## 3. Measurement-Device Independency Analysis Method

If the task of a quantum cryptographic protocol is completed under the MDI assumptions, the protocol is measurement-device-independent. The task of CVQHS is to verify the identities of different data sources at a low error rate. Thus, in the measurement-device analysis of the CVQHS scheme, we can calculate the upper bound of the error rate. If the upper bound is negligible under the MDI assumptions, the CVQHS scheme is measurement-device-independent.

The upper bound of the error rate is the error rate under the worst case when an attacker can carry out any possible attack. Thus, we will find out the optimal attack model and calculate the error rate under the model.

### 3.1. Attack Model

**Figure 2:**
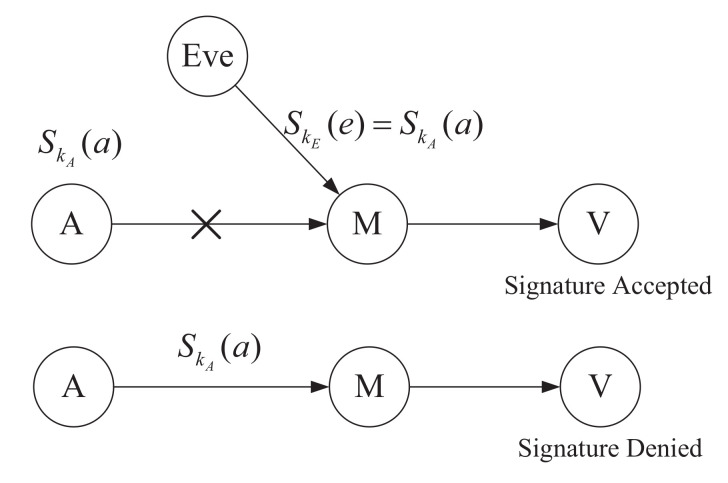
Possible errors in CVQHS.

Considering all possible cases which are shown in Figure 2, the error rate is equal to the probability of a forged signature passing verification plus the probability of a legal signature being denied.

Obviously, the probability of a legal signature being denied is only affected by noise. Thus, we only consider the attack model of the case that an attacker tries to forge a signature. In the CVQHS scheme, when an attacker Eve has secret keys and is able to prepare quantum states which are entangled with those at honest signers, it can forge a signature that can pass verification.

Throughout the CVQHS scheme, only the aggregator *M* and the verifier *V* use measurement devices. Here, we assume the measurement devices controlled by *V* are trusted because the protocol will be extremely inefficient and meaningless if the verifier is dishonest. Thus, the MDI assumptions only apply to the measurement devices controlled by *M*, namely a 50:50 BS and two homodyne detectors which are used to perform Bell detection, and a 50:50 BS for mixing two quantum signatures. According to Assumption (1), Eve is able to tamper and forge the results of Bell detection and the mixtures of quantum signatures at the combining phase. Thus, Eve can forge a quantum signature that can pass verification as long as it obtains the pre-shared secret keys. Thus, the security of the CVQHS scheme is guaranteed by the secrecy of secret keys. The probability of a forged signature passing verification is equal to the probability of Eve obtaining secret keys. At this point, the complicated attack model which contains forgery is simplified as a simple eavesdropping model.

According to Assumption (2), Eve is able to eavesdrop all quantum channels by any means. From the perspective of an attacker’s ability, eavesdropping can be divided into three types, namely coherent attack, collective attack and individual attack. Coherent attack is the most general attack by which an attacker can perform joint quantum operations and joint measurement to all quantum states sent via quantum channels. The proof of security against coherent attack is the strictest proof for security, but the model of coherent attack cannot be effectively parameterized. A common approach is to extend the security against collective attack to coherent attack by using the exponential de Finetti theorem [20]. Collective attack is a special case of coherent attack, where an attacker can only perform quantum operations individually on each quantum state.

Fortunately, analysis shows that the security bound under coherent attack is the same as that under collective attack for QKD protocols [21]. This result can be applied to CVQHS because a signature in the scheme is a single quantum state. The quantum states in a quantum channel are not correlated, so introducing correlations to them by performing joint operations will not help the attacker obtain more information. Therefore, we can analyze the security against collective attack.

### 3.2. Probability of a Forged Signature Passing Verification

At the first step of the setup phase, the signers and the verifier share secret keys. Assume they use a MDI-QKD protocol in this step; then, Eve can only obtain the secret keys by eavesdropping the quantum channels. The information on the secret keys that Eve can obtain is the mutual information I(k:E), where $k=(k_{1},k_{2})$ denotes the secret keys and *E* is the quantum system of Eve. The larger the mutual information I(k:E) is, the more information Eve can obtain. When I(k:E)=H(k), Eve can recover the secret keys accurately. The upper bound of I(k:E) is usually used to estimate the security of a protocol.

According to the symmetry of CVQHS, we only need to calculate the upper bounds of $I(k_{A_{1}}:E)$ and $I(k_{A_{2}}:E)$. According to quantum information theory, it is known that $I\left(k_{A_{1}}:E\right)\leq \chi \left(k_{A_{1}}:E\right)$, where $\chi (k_{A_{1}}:E)$ is the Holevo bound [22]. It can be calculated that $\chi \left(k_{A_{1}}:E\right)=S\left({\hat{\rho }}_{E}\right)‒S\left({\hat{\rho }}_{E}|k_{A_{1}}\right)$ under collective attack, where $S\left({\hat{\rho }}_{E}|k_{A_{1}}\right)=\int p(k_{A_{1}})S\left({\hat{\rho }}_{E|k_{A_{1}}}\right)dk_{A_{1}}$ and ${\hat{\rho }}_{E}$ is the quantum system of Eve. According to assumption (1) aforementioned in Section 2.1, Eve can purify the whole quantum system, so $\chi \left(k_{A_{1}}:E\right)=\chi \left(k_{A_{1}}:{\hat{\rho }}_{1^{\prime }2^{\prime \prime }3^{\prime }4^{\prime \prime }}\right)$, where ${\hat{\rho }}_{1^{\prime }2^{\prime \prime }3^{\prime }4^{\prime \prime }}=|\alpha_{1}^{\prime }\rangle |\alpha_{2}^{\prime \prime }\rangle |\alpha_{3}^{\prime }\rangle |\alpha_{4}^{\prime \prime }\rangle$. Because $|\alpha_{1}^{\prime }\rangle$ and $|\alpha_{3}^{\prime }\rangle$ are independent of the secret keys, their entropy will be offset during subtraction. Thus, $S\left({\hat{\rho }}_{E}\right)‒S\left({\hat{\rho }}_{E}|k_{A_{1}}\right)=S\left({\hat{\rho }}_{2^{\prime \prime }4^{\prime \prime }}\right)‒S\left({\hat{\rho }}_{2^{\prime \prime }4^{\prime \prime }}|k_{A_{1}}\right)$, where ${\hat{\rho }}_{2^{\prime \prime }4^{\prime \prime }}=|\alpha_{2}^{\prime \prime }\rangle |\alpha_{4}^{\prime \prime }\rangle$.

The quantum states in the CVQHS scheme are Gaussian states, whose von Neumann entropy can be calculated based on their covariance matrices. Assume the original entangled states prepared by the aggregator have the same density matrix, i.e., $\rho_{12}=\rho_{34}=\rho_{\mathrm{in}}$. Their covariance matrix is

$$V_{in}=\left(\begin{array}{cc} VI & \sqrt{V^{2}‒1}diag\left(1,‒1\right)\\ \sqrt{V^{2}‒1}diag\left(1,‒1\right) & VI \end{array}\right),$$

where V=cosh2r is the variance of two-mode squeezed states. Assume the quantum channels are modeled as

(3) $$\left|\alpha \rangle \to \right|\sqrt{\tau }\alpha +\sqrt{1‒\tau }\alpha_{N}\rangle ,$$

where τ(0<τ<1) is transmissivity and $|\alpha_{N}\rangle =|x_{N}+ip_{N}\rangle$ is thermal noise. Assume thermal noise in each quantum channel is independently and identically distributed and their quadratures follow Gaussian distribution: $x_{N},p_{N}∼N\left(0,V_{N}\right)$.

After |α2〉 and |α4〉 are transmitted twice via noisy quantum channels, the covariance matrix becomes

$$V_{in}^{\prime }=\left(\begin{array}{cc} V_{1}I & \sqrt{{V_{1}}^{2}‒1}diag\left(1,‒1\right)\\ \sqrt{{V_{1}}^{2}‒1}diag\left(1,‒1\right) & V_{1}I \end{array}\right),$$

where $V_{1}=\tau^{2}V+\left(\tau +1\right)V_{N}$.

After entanglement swapping, the covariance matrix of ${\hat{\rho }}_{2^{\prime }4^{\prime }}=|\alpha_{2}^{\prime }\rangle |\alpha_{4}^{\prime }\rangle$ is

$$V_{2^{\prime }4^{\prime }}=\frac{1}{2V_{1}}\left(\begin{array}{cc} diag({V_{1}}^{2}+1,{V_{1}}^{2}+1) & diag({V_{1}}^{2}‒1,‒{V_{1}}^{2}+1)\\ diag({V_{1}}^{2}‒1,‒{V_{1}}^{2}+1) & diag({V_{1}}^{2}+1,{V_{1}}^{2}+1) \end{array}\right).$$

Then, $|\alpha_{2}^{\prime }\rangle$ and $|\alpha_{4}^{\prime }\rangle$ are mixed at a 50:50 beam splitter, outputting $|\alpha_{2}^{\prime \prime }\rangle$ and $|\alpha_{4}^{\prime \prime }\rangle$. Beam splitter is a Gaussian operator, which does not change the von Neumann entropy of a quantum system. Thus, the von Neumann entropy of ${\hat{\rho }}_{2^{\prime \prime }4^{\prime \prime }}$ can be calculated based on $V_{2^{\prime }4^{\prime }}$.

$S({\hat{\rho }}_{2^{\prime \prime }4^{\prime \prime }}|k_{A_{1}})$ is the von Neumann entropy of ${\hat{\rho }}_{2^{\prime \prime }4^{\prime \prime }}$ when $k_{A_{1}}$ is given. It can be calculated based on a new covariance matrix

$$V_{2^{\prime }4^{\prime }|k_{A_{1}}}=\frac{1}{2V}\left(\begin{array}{cc} diag({V^{\prime }}^{2}+1,{V^{\prime }}^{2}+1) & diag({V^{\prime }}^{2}‒1,‒{V^{\prime }}^{2}+1)\\ diag({V^{\prime }}^{2}‒1,‒{V^{\prime }}^{2}+1) & diag({V^{\prime }}^{2}+1,{V^{\prime }}^{2}+1) \end{array}\right),$$

where $V^{\prime }=V_{1}‒V_{k_{A_{1}}}$.

Simple calculation shows that $I(k_{A_{1}}:E)=0$, which means Eve cannot obtain any information on $k_{A_{1}}$. Similarly, we can calculate that $I(k_{A_{2}}:E)=0$. Thus, Eve cannot obtain any information on the pre-shared secret keys between the signers and the verifier. The probability of a forged signature passing verification is the probability of Eve guessing the exact secret keys, which is negligible.

In the above theoretical analysis, we only considered the case of collective attack, which is proved to be the optimal attack model. In fact, simulation or experiment considering more complex scenarios can be conducted to verify our calculation results in future works. It will be much easier to obtain the error rate for complex scenarios such as coherent attack and forgery, which involve complex modeling and calculation in theoretical analysis and cannot be efficiently parameterized [21]. Special attack models may be also implemented to discuss how parameters affect the result of CVQHS.

### 3.3. Probability of a Legal Signature Being Denied

In the CVQHS scheme, if the deviation between the value calculated from a signature and the value calculated from pre-shared messages is larger than certain verification threshold, the signature will be denied by the verifier. The deviation can be caused by an attacker or noise. Here, it is assumed that the verifier receives a signature that is generated by a legal signer and not tampered by an attacker. Thus, the probability only depends on noise.

A verification threshold $H_{th}$ in a noisy environment is given in Ref. [19], which is equal to the variance of $x_{V}‒\tau x_{V}^{\prime }$. In the verification phase, the verifier compares ${\left(x_{V}‒\tau x_{V}^{\prime }\right)}^{2}$, ${\left(p_{V}‒\tau p_{V}^{\prime }\right)}^{2}$ and $H_{th}$. If ${\left(x_{V}‒\tau x_{V}^{\prime }\right)}^{2}>H_{th}$ or ${\left(p_{V}‒\tau p_{V}^{\prime }\right)}^{2}>H_{th}$, it will deny the signature. Denote $x_{V}‒\tau x_{V}^{\prime }$ as a random variable *X* whose first and second moments are EX=0 and $DX=H_{th}$. Thus, the probability of a legal signature being denied is

$$\begin{array}{cc} P(X^{2}>H_{th}) & =P(X^{2}>DX)\\ & =P(|X|>\sqrt{DX}) \end{array}$$

Since *X* is a linear combination of quadratures, secret keys and classical messages, it follows the Gaussian distribution. According to the property of Gaussian distribution, $P(X^{2}>H_{th})\approx 0.32$. Thus, the probability of a legal signature being denied is 0.32.

By adding up two probabilities in Section 3.2 and Section 3.3, we can conclude that the upper bound of the error rate of the CVQHS scheme is 0.32 when all measurement devices are untrusted. Although 0.32 is not negligible, the probability of correctly verifying the identities is twice of error rate. Thus, the CVQHS scheme is deemed to be measurement-device-independent.

### 3.4. Discussion

Firstly, we discuss how the parameters of the CVQHS scheme affect the error rate.

The calculation of probability of a forged signature passing verification involves three parameters, namely the variance *V* of two-mode squeezed states, the transmissivity τ of quantum channels, and the variance $V_{N}$ of thermal noise of quantum channels. According to calculation result, the probability is always 0 provided *V* is nonzero, which means an attacker cannot obtain the pre-shared secret keys as long as the entangled states are properly prepared and not collapsed before being used for generating quantum signatures. Noisy quantum channels do not have any influence on the probability of a forged signature passing verification. It is the randomness of quantum states that prevents the pre-shared secret keys from being leaked during transmission.

The calculation of probability of a legal signature being denied involves the values of both quadratures of entangled states, pre-shared secret keys, the transmissivity and the variance of thermal noise of quantum channels, and the verification threshold. In the calculation, the parameters follow Gaussian distribution so the probability can be easily obtained. The probability is influenced by the verification threshold $H_{t}h$. If $H_{t}h$ is larger, the will decrease but it will be easier for a forged quantum signature to pass verification. If $H_{t}h$ is smaller, the probability will increase. Thus, the verification should be carefully set in order to lower the error rate.

Secondly, we discuss the application of our analysis method. Our analysis method can be summarized in the following three steps:

Step 1. Analyze the objective of the protocol and find the parameter that can be used to decide whether the protocol has completed its task.

Step 2. Analyze the topology and the communication pattern of the protocol to obtain a simplified attack model, which may be a sufficiently studied attack.

Step 3. Calculate the parameter under the attack model to judge the measurement-device independency of the protocol.

In our analysis procedure, the parameter is the upper bound of error rate and the attack model can be simplified as collective attack. Although we only analyze the CVQHS scheme, the analysis method can be applied to other CVQDS protocols by means of calculating the same parameter under a similar attack model.

Concretely, the objective of a CVQDS protocol is to verify the identity of a data source, which is the same as the CVQHS scheme. Thus, at Step 1, the parameter will be the upper bound of error rate as well. From the perspective of verification results, errors can be classified into two types. The first type of error is the case where a tampered or forged quantum signature passes verification. The second type of error is the case where a legal quantum signature which is not tampered by attackers gets denied by the verifier. To calculate the error rate, we should respectively construct models for the two type of errors. The first type of error usually evolves attackers so we should construct an attack model. The second type of error is caused by noise so we should also construct a model for noisy quantum channels.

Constructing an attack model at Step 2 is the key step of our MDI analysis method. The most effective way of attack can be found by means of applying MDI assumptions to the protocol. Attack models may be different for different CVQDS protocols if the protocols have different network topologies and communication patterns. Since most of the CVQDS protocols do not involve an untrusted aggregator, we believe attack models for CVQDS protocols will be simpler than the CVQHS scheme. Furthermore, it seems that the attack model of a CVQDS protocol can often become an eavesdropping model because it is necessary for an attacker to obtain secret keys. After simplification, the calculation process at Step 3 will be similar to our calculation.

The above analysis procedure seems to be a general formalism for analyzing measurement-device independency. In this procedure, the key point of analyzing a protocol is to find an appropriate parameter and constructing an attack model. For a complicated protocol carried out in a large-scale network, it may have several tasks that affect each other and each task is completed by several nodes. It will be difficult to find an appropriate parameter at Step 1. In addition, unintended entanglement among different nodes will not only affect the quantum states transmitted between two legal nodes in an unexpected way, but also increase the complexity of analysis and calculation. It will be difficult to construct an attack model that is simple enough for calculation. Thus, MDI analysis method of quantum cryptographic protocols except CVQDS protocols still needs to be explored.

## 4. Conclusions

In this paper, we analyze the measurement-device independency of continuous-variable quantum digital signature. According to the objective of CVQDS, we proposed that a CVQDS protocol is measurement-device-independent if its error rate is negligible on condition that all measurement devices are untrusted. Concretely, we take a continuous-variable quantum homomorphic signature protocol as an example. The error rate of the CVQHS scheme is equal to the probability of a forged signature passing verification plus the probability of a legal signature being denied. In the analysis procedure, we constructed an attack model in order to calculate the error rate. The attack model was simplified as collective attack by means of applying MDI assumptions to the protocol. Calculation was also simplified by using an advantage of Gaussian states, i.e., the von Neumann entropy of a Gaussian state can be calculated from its first and second moments. Calculation results show that the error rate is 0.32 so that the CVQHS scheme is deemed to be measurement-device-independent. Although we only analyzed the measurement-device independency of the CVQHS scheme, our analysis can be summarized in three steps and applied to other CVQDS protocols. Whether this approach is a general formalism for analyzing the measurement-device independency of all quantum protocols is still an open question and will be discussed in future works.

## Abbreviations

The following abbreviations are used in this manuscript:

QKD	Quantum Key Distribution
DI	Device-Independent
MDI	Measurement-Device-Independent
DI-QKD	Device-Independent Quantum Key Distribution
MDI-QKD	Measurement-Device-Independent Quantum Key Distribution
CV-MDI-QKD	Continuous-Variable Measurement-Device-Independent Quantum Key Distribution
CVQDS	Continuous-Variable Quantum Digital Signature
CVQHS	Continuous-Variable Quantum Homomorphic Signature
BS	Beam Splitter
